# Supplementary material for: A three-dimensional finite element analysis on the effects of implant materials and designs on periprosthetic tibial bone resorption
Source: PLoS One. 2021 Feb 10;16(2):e0246866. doi: 10.1371/journal.pone.0246866 (PMC7875353; doi:10.1371/journal.pone.0246866)
Supplement: Attachment — Submitted filename: Author response (review 1st).docx [file pone.0246866.s001.docx]

Author response

Changes in manuscript are marked in red color

Journal Requirements:

When submitting your revision, we need you to address these additional requirements.

1. Please ensure that your manuscript meets PLOS ONE's style requirements, including those for file naming. The PLOS ONE style templates can be found at

https://journals.plos.org/plosone/s/file?id=wjVg/PLOSOne_formatting_sample_main_body.pdf and

https://journals.plos.org/plosone/s/file?id=ba62/PLOSOne_formatting_sample_title_authors_affiliations.pdf

-> We have reviewed the sample of the manuscript and authors affiliation form.

2. Please provide additional details regarding patient consent for the CT images to be used for building the proximal tibia model in this research. In the ethics statement in the Methods and online submission information, please ensure that you have specified (1) whether consent was informed and (2) what type you obtained (for instance, written or verbal, and if verbal, how it was documented and witnessed).

-> We have added the informed consent from the patient on the method section. (Line 151-153)

General Suggestions:

1. Clearly point out that this is a N=1 virtual perturbation study in the abstract and the introduction as well.

-> We totally agreed with the reviewer’s comment. we have added what the reviewer commented. (Line 25-26, Line 86-87)

2. Consider the recommendations given in

Erdemir, A., Guess, T. M., Halloran, J., Tadepalli, S. C., & Morrison, T. M. (2012). Considerations for reporting finite element analysis studies in biomechanics. Journal of biomechanics, 45(4), 625-633.

Viceconti, M., Olsen, S., Nolte, L. P., & Burton, K. (2005). Extracting clinically relevant data from finite element simulations. Clinical Biomechanics, 20(5), 451-454.

- > We thank the reviewer for the recommendation.

3. Are the bone and implant geometries available (for further research comparisons)?

-> We totally agreed with the reviewer’s comment. As the reviewer’s comment, we have added geometries on table 1. (Line 141-142)

4. Please list the material properties in detail: Young's modulus, Poisson number, etc.

-> We totally agreed with the reviewer’s comment. As the reviewer’s comment, we have added material properties on table 2. (Line 143-144)

5. Please elaborate on the mesh configuration of bone and implants.

-> We totally agreed with the reviewer’s comment. As the reviewer’s comment, we have added mesh convergence test (Line 133-137) and mesh configuration on table 3. (Line 145-146)

6. Could you quantify the structural stiffness of the different implants (cross-sectional area, area moments, etc.)?

-> We totally agreed with the reviewer’s comment. As the reviewer’s comment, we have added geometries on table 1. (Line 141-142)

7. Assumptions such as full bonding of interfaces should be justified in the text.

-> Thank you for your commenting. As the reviewer’s comment, we clarified that the components and cement were full bonded and not to be displaced. (Line 129-130)

8. Check English spelling.

-> Thank you for your commenting. We have carefully reviewed the manuscript.

Major concerns:

1. Reporting of modeling details is insufficient, this includes:

a) mesh configuration (number elements, nodes, element types, mesh convergence)

-> We totally agreed with the reviewer’s comment. As the reviewer’s comment, we have added mesh convergence test (Line 133-137) and mesh configuration on table 3. (Line 145-146)

b) boundary conditions (only loading is described without describing application in detail [point loads?], displacement constraints are missing)

-> We totally agreed with the reviewer’s comment. As the reviewer’s comment, we clarified that the components and cement were full bonded and not to be displaced. (Line 129-130). The axial force was applied on the point of proximal tibial model. (Line 149-151).

c) geometries are only partially described, missing structural stiffness, some size dimensions (name implant type and size?)

-> We totally agreed with the reviewer’s comment. As the reviewer’s comment, we have added geometries on table 1. (Line 141-142)

d) Simulation software (solver, post-processing) is not clear

-> We totally agreed with the reviewer’s comment. As the reviewer’s comment, we have added what we used in post-processing program. (Line 137)

e) Validation was not performed, this should be justified

-> We totally agreed with the reviewer’s comment. We also thought that validation was important issue in the virtual perturbation study. However, as the study was performed using clinical images of CT, validation was difficult to be done. Therefore, we selected the convergence test as the tool of justifying. Because the error in strain was less than 3% in our study, we considered that the findings of our study was reasonable. (Line 133-137)

f) Material properties should be listed in detail and not only referenced

-> We totally agreed with the reviewer’s comment. As the reviewer’s comment, we have added material properties on table 2. (Line 143-144)

Detailed comments

l.98 "heterogenetic"? Did you mean heterogeneous?

-> We totally agreed with the reviewer’s comment. As the reviewer’s comment, we clarified what we wanted to express in the word ‘hetrogenetic’. Because the proximal tibia was consisted of cortical and cancellous bone, the structural material of the proximal tibia was not homogenous. To reflect the characteristics of in-vivo, the proximal tibia model was reconstructed using CT images. (Line 102-105).

l.112-3 is redundant with l.128-30, although a reference was added, are this in-vivo measured loads?

-> We totally agreed with the reviewer’s comment. As the reviewer’s comment, we have deleted the redundant sentences.

l.142-3 is redundant with l.149ff;

Figure legends do not add much information, please rather describe the figures in more detail. Add additional information directly on the figures, by this you can avoid confusion.

-> We totally agreed with the reviewer’s comment. As the reviewer’s comment, we have deleted the redundant sentences.

l.156 should be in the discussion, please just list the results first, before summarizing it

-> We totally agreed with the reviewer’s comment. As the reviewer’s comment, we have re-positioned that in the discussion section. (Line 272-273)

l.216 Please rephrase: “Studies have reported medial periprosthetic tibial bone resorption after TKA using implants with stiffer materials

-> We totally agreed with the reviewer’s comment. As the reviewer’s comment, we have changed. (Line 236-237)

l.217-220 Do not forget the exchanging loading in an in-vivo situation. Although you considered a realistic 60/40 ratio, this is not always true and change according to the activities.

-> We totally agreed with the reviewer’s comment. we have added what the reviewer’s comment to our one of the limitations. (Line 303-305)
